# Potential of Microneedle Systems for COVID-19 Vaccination: Current Trends and Challenges

**DOI:** 10.3390/pharmaceutics14051066

**Published:** 2022-05-16

**Authors:** Jasmin Hassan, Charlotte Haigh, Tanvir Ahmed, Md Jasim Uddin, Diganta B. Das

**Affiliations:** 1 Drug Delivery & Therapeutics Lab, Dhaka 1212, Bangladesh; jasmin.hasan10@gmail.com (J.H.); tanvirahmedpranto558@gmail.com (T.A.); 2 Department of Chemical Engineering, Loughborough University, Epinal Way, Loughborough LE11 3TU, UK; c.r.haigh-17@student.lboro.ac.uk; 3 Faculty of Engineering and Science, University of Greenwich, Chatham Maritime, Kent ME4 4TB, UK; 4 Department of Pharmacy, Brac University, 66 Mohakhali, Dhaka 1212, Bangladesh

**Keywords:** COVID-19 vaccine delivery, dissolvable microneedles, immunogenicity, mass vaccination

## Abstract

To prevent the coronavirus disease 2019 (COVID-19) pandemic and aid restoration to prepandemic normality, global mass vaccination is urgently needed. Inducing herd immunity through mass vaccination has proven to be a highly effective strategy for preventing the spread of many infectious diseases, which protects the most vulnerable population groups that are unable to develop immunity, such as people with immunodeficiencies or weakened immune systems due to underlying medical or debilitating conditions. In achieving global outreach, the maintenance of the vaccine potency, transportation, and needle waste generation become major issues. Moreover, needle phobia and vaccine hesitancy act as hurdles to successful mass vaccination. The use of dissolvable microneedles for COVID-19 vaccination could act as a major paradigm shift in attaining the desired goal to vaccinate billions in the shortest time possible. In addressing these points, we discuss the potential of the use of dissolvable microneedles for COVID-19 vaccination based on the current literature.

## 1. Introduction

One of the greatest public health threats faced by humanity in this century is the coronavirus disease 2019 (COVID-19) pandemic caused by the severe acute respiratory syndrome coronavirus 2 (SARS-CoV-2) [1,2,3]. While the entire world is yearning for relief from this pandemic, a ray of hope was foreseen via the approval of several COVID-19 vaccines by the Food and Drug Administration (FDA) [4,5] and other national agencies (e.g., the UK’s the Medicines and Healthcare products Regulatory Agency). However, the effectiveness of the vaccination programs and the global outreach of the vaccines for the mass population worldwide are major areas of concern [2,4,5] given the need to vaccinate billions of people in the shortest period of time. This requires the immense efficiency of the vaccination programs [6,7,8]. The vaccines require special storage conditions to remain viable during transportation and distribution [9,10,11]. The delivery of the vaccines can only be done via trained professionals using conventional vaccine delivery using hypodermic syringes [12,13]. However, the scarcity of trained professionals and the absence of adequate dosages are two of the key barriers to attaining mass vaccination [14,15]. Moreover, the vaccination programs would mean mass gatherings at specific places, which is risky, since they have the potential to facilitate the quick spread of the disease [16,17,18].

In addition, mass vaccination using hypodermic syringes will produce a massive amount of biowaste, which would create another area of concern for effective waste management [13,19,20]. In this scenario, we have to think about alternatives which would make the vaccination program much more efficient and accessible [21,22,23]. The use of novel vaccine delivery could be a potential solution to this critical challenge [24,25,26]. The use of microneedle vaccine delivery mechanisms would allow the vaccines to be delivered in a painless manner, ensuring the controlled release of drugs via a dissolvable microneedle [27,28,29,30].

The use of dissolvable microneedle patches would improve the dosing accuracy, ensuring the precise delivery of the vaccines [31,32,33].

The use of biodegradable microneedles for transdermal immunization is a fast-developing topic of study and application [34,35,36]. One of the main reasons why most individuals refuse to be vaccinated is their fear of painful needles [37,38,39]. As a result, creating a pain-free technique of immunization utilizing microneedles has been a major research challenge [40,41,42].

Microneedles contain arrays of micron-sized needles that deliver molecules across the skin without causing discomfort [43,44,45]. Microneedles have a number of benefits over traditional immunization methods (summarized in Table 1), such as intramuscular and subcutaneous injections, aside from the fact that they are painless [46,47,48]. Microneedle vaccinations elicit a strong immune response because the needles, which range in length from 25 to 1000 µm, may effectively transport the vaccine to the epidermis and dermis, which contain a large number of Langerhans and dendritic cells [36,49,50]. The microneedle array resembles band-aid patches and provides the benefits of cold-chain storage avoidance and self-administration flexibility [49,50,51]. Microneedles have the benefit of slowing the release of vaccination antigens [52,53,54]. Vaccine components in microneedles might be in solution or suspension, coated in nano or microparticles, or based on nucleic acid [55,56]. Because of the combined benefits of particulate vaccinations and pain-free vaccination, the use of microneedles to administer particle-based immunizations is growing rapidly [57,58,59]. The future of microneedle-based vaccines is bright, but certain constraints, such as dosage insufficiency, stability, and sterility, must be addressed before microneedles may be successfully used for vaccine delivery [60,61,62]. This article summarizes the current developments in COVID-19 medicaments and vaccine delivery in accordance with the field of microneedle-based immunization [61,63]. Table 1 shows a brief comparison between the limitations of needle vaccination and microneedle vaccine delivery systems. It lists the comparative differences between syringe and microneedle vaccination and shows how it is a viable and better alternative for mass COVID-19 vaccination. Given the aim of this review paper, we restrict ourselves to reviewing the literature of microneedle-based vaccine delivery methods.

### 1.1. Immunological Aspects of COVID-19 Medicaments

**Figure 1:**
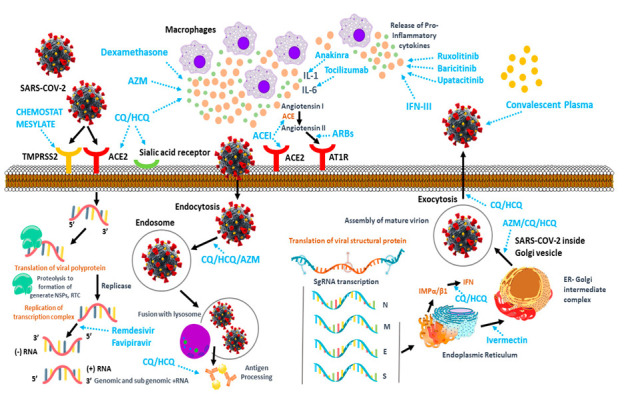
SARS-CoV-2 replication process and possible therapeutic targets (reproduced from [71]).

Scientists all over the world are still searching to find an effective cure for COVID-19, which might end the pandemic so that the world can go back to normalized pre-COVID-19 life [63,69,70]. Unfortunately, the search is still ongoing and will continue until a potent treatment is discovered [8,9,10]. Various therapeutic strategies are under development and are being tested worldwide [11,12]. The outcome of the treatment procedure depends on the host humoral response and the cellular immunity of the patient due to COVID-19 infection [13,14,15]. Table 2 shows the induced immunopathology of the SARS-CoV-2 virus along with the humoral response in humans. Responses from previous SARS-CoV-2 infections could act as key determinants in the development of the therapeutics, since the infection causes the production of anti-SARS-CoV-2 antibodies [16,17,18]. The antibodies produced via the immune response from the infection limits replication through the neutralization of the virus inside the body and plays a major role in controlling the disease [13,20,21]. This mechanism might also contribute to the development of COVID-19 pathogenesis due to the involvement of antibody-dependent enhancement [22,23]. Approaches such as convalescent plasma and monoclonal antibodies have enabled expeditious development in research associated with the treatment of COVID-19 in terms of distinguishing the widely varied clinical features of antibody responses in SARS-CoV-2-infected patients worldwide [24,25,26]. Clinical outcomes from numerous COVID-19 vaccine candidates have been determined, as well as the collection and characterization of a wide panel of monoclonal neutralizing antibodies and early clinical testing [27,28,30]. Figure 1 illustrates the replication process of SARS-CoV-2 and lists the therapeutic targets during the replication process.

### 1.2. Structural Considerations of Coronavirus in Vaccine Development

SARS-CoV-2 is a member of the Coronaviridae family’s subgroup, which includes four species: α, β, γ, and δ. SARS-CoV and Middle East respiratory syndrome (MERS-CoV) are two more extremely deadly viruses in the Coronaviridae family that produced epidemics in 2002–2003 and 2013–present, respectively [7,8,11,13]. The spike (S), envelope (E), membrane (M), and nucleocapsid (N) structural proteins, as well as at least six auxiliary proteins (3a, 6, 7a, 7b, 8, and 10), are all encoded by the SARS-CoV-2 RNA genome [13,14,15,16]. The S protein, which consists of the S1 subunit, S2 subunit, transmembrane, and cytoplasmic domains, infects host cells [22,24,25]. The N-terminal domain (NTD), receptor-binding domain (RBD), subdomain 1 (SD1), and subdomain 2 (SD2) make up the S1 subunit (SD2) [27,28,30]. The RBD of SARS-S1 CoV-2’s subunit interacts with the cellular receptor angiotensin-converting enzyme 2. (ACE2) [83,84,85]. Virus entrance is mediated by CD147. SARS-CoV-2 enters the cytoplasm with its RNA genome and undergoes intracellular replication cycles before being discharged by exocytosis to infect new host cells. COVID-19 patients have a significant morbidity and death rate, which necessitates the rapid development of efficient preventive and treatment measures [82,86,87]. Pneumonia is the most common complication of SARS-CoV-2 infection. COVID-19 individuals who are severely sick or dangerously ill have additional organ damage [88,89]. COVID-19 initiation, amplification, and consummation are three separate stages that patients go through [90,91,92]. Rapid viral replication and the early production of dominant chemokines define the beginning stage [74,93,94]. If the viral infection is not effectively suppressed by the host’s humoral defenses, the patient enters the multiplication stage after developing both humoral and cellular immunity, during which, they produce more inflammatory mediators and recruit a large number of inflammatory cells to increase immunopathological processes [95,96,97]. COVID-19 victims eventually die as a result of persistent elevations in inflammatory mediators and extensive organ damage throughout the culmination stage [98,99,100]. Antibody-mediated humoral immune responses to SARS-CoV-2 infection are critical in the progression of COVID-19 illness [101,102]. In addition, outcomes from clinical research employing convalescent plasma and intravenous immunoglobulins (IVIG) to treat COVID-19 patients with passive antibody therapy have been published [103,104,105]. A vast number of neutralizing monoclonal antibodies have been identified, with some of them having undergone clinical testing [106,107,108]. Clinical effectiveness data from numerous vaccination studies have been published, which are significant [109,110,111]. Table 3 shows the various technologies used for the development of COVID-19 vaccines.

**Figure 2:**
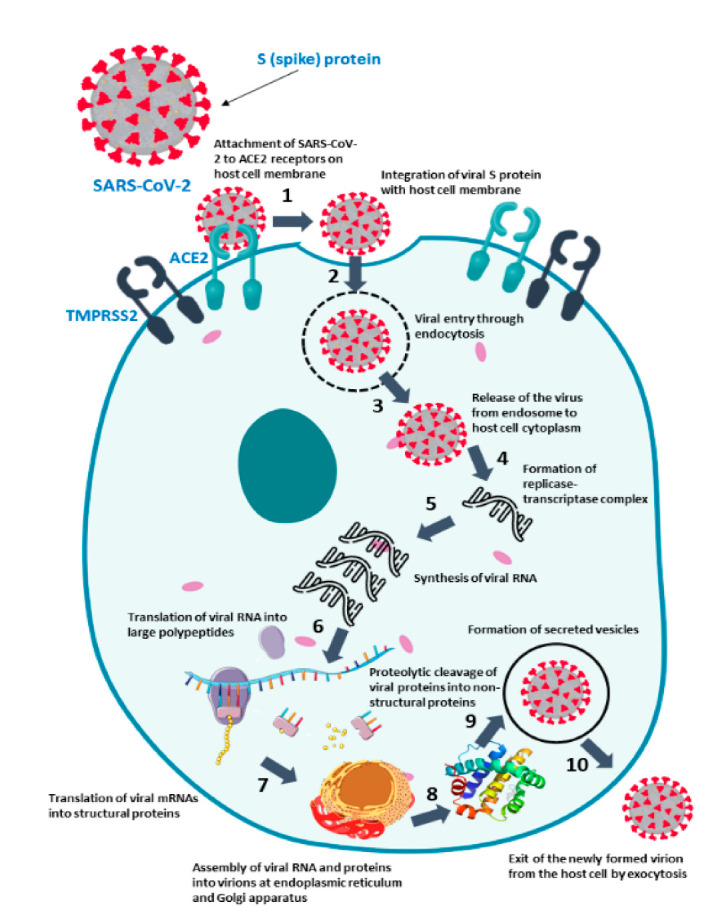
Various (numbered) stages involved in COVID-19 pathogenesis that might be used as targets for targeted treatment and vaccine development (reproduced from [155]).

Coronaviruses are single-stranded RNA viruses with an enclosed surface glycoprotein spike that facilitates receptor binding and cell penetration during infection [112,113]. Because of its functions in receptor binding and membrane fusion, the spike protein is a promising vaccination antigen [114,115,116]. Apart from entire virion-inactivated vaccines, almost all producers are targeting spike protein as an antigen [117,118,119]. Instead of incorporating the entire pathogen, new-generation vaccinations, such as recombinant protein vaccines and vector-based vaccines, only include a particular antigen or antigens from the disease, providing a superior safety profile [120,121,122]. Detailed knowledge of the pathogen’s structure and immunopathogenesis is required for the development of an effective new-generation vaccine [123,124]. Depending on the carrier of the antigen, the new-generation COVID-19 vaccines may be divided into recombinant protein vaccines and vector vaccines (for instance, mRNA vaccines, plasmid DNA vaccines, viral vector vaccines, and bacterial vector vaccines) [125,126,127]. The structural and pathobiology characteristics of the SARS-CoV-2 virus were used to choose the target antigen for new-generation vaccination [128,129,130]. The SARS-CoV-2 genome is a single-stranded, positive-sense RNA. The S proteins are found on the virus’s outer surface and can bind to ACE2 on the cell surface, allowing for receptor-mediated viral endocytosis [131,132,133]. Animal models that express human ACE2 counterparts should be utilized in challenge experiments to evaluate vaccination effectiveness, according to the ACE2-dependent mechanism [134,135]. Although the SARS-CoV S protein can employ CD209 and CD209L as alternative receptors, it is unknown if SARS-CoV-2 can as well [136,137,138]. Most COVID-19 vaccine candidates employ the S protein as the antigen since it plays such an important part in the virus’s life cycle [139,140]. The use of RNA for vaccine development is a game-changing technique that requires the adoption of a suitable delivery method to increase the oligonucleotide’s intracellular stability, and hence, translatability [141,142] The antigen of interest is encoded by non-replicating mRNA vaccines, but self-amplifying RNA leads to the translation of both the antigen and the viral replication machinery, allowing intracellular RNA amplification and protein expression [143,144,145]. Figure 2 illustrates the different stages of the pathogenesis of COVID-19 and possible targets for vaccine development.

While it is critical to expedite the development of these immune-mediated treatments, it is also vital to remember that there are still many unanswered issues about SARS-CoV-2 infection and its influence on host immunity [114,146]. On the one hand, this enhanced information is essential for elucidating the many processes of the host immune response involved in viral neutralization and/or the elimination of infected cells [147,148,149]. On the other hand, it will also forecast the outcome when used at a large scale in the targeted demographic [150,151,152]. As a result, the effect of gender and age on the virus’s ability to modulate host immune response, as well as the impact of SARS-CoV-2 infection on the immunity of patients with chronic diseases such as diabetes, hypertension, and chronic obstructive pulmonary disease (COPD), must be fully addressed [153,154].

### 1.3. Distribution Concerns of COVID-19 Vaccines

Prior to the COVID-19 pandemic, researchers did not seem to pay regular attention to the storage temperature for mRNA vaccine candidates that were under the development process. Small quantities were often frozen at −80 °C and then thawed and injected as needed [162,164,165]. Table 4 shows the current mRNA COVID-19 vaccine stability profile, dose, and dosing schedule.

**Figure 3:**
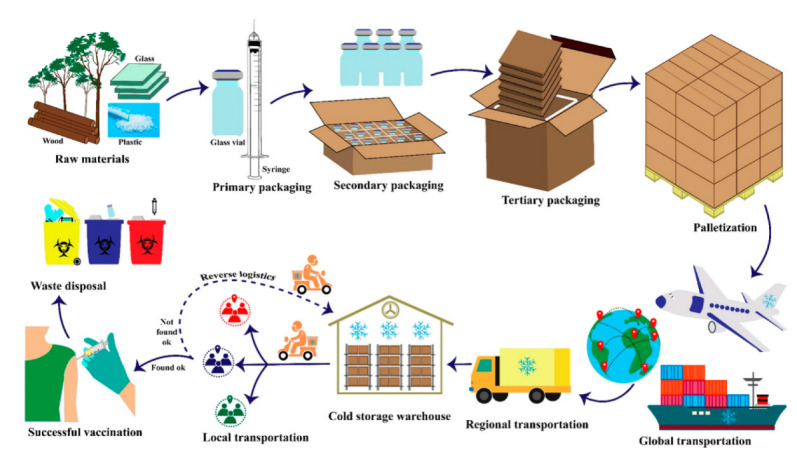
The coronavirus disease vaccine is packaged and distributed in various levels to ensure cold storage throughout the supply chain for a successful vaccination campaign (taken from) [190].

In addition to the rising clinical promise of mRNA-based COVID-19 vaccines, there was a growing concern that storage, transport, and administration under these conditions would pose a significant problem when hundreds of millions (if not billions) of doses were to be distributed globally [170,171]. It is critical to understand that the entire undamaged mRNA molecule is required for the vaccine’s effectiveness [172,173,174]. Even a small degradation event anywhere along an mRNA strand might significantly delay or halt that strand’s normal translation performance, resulting in inadequate antigen expression [175,176,177]. Therapeutic proteins and protein antigens, on the other hand, may undergo numerous chemical degradation processes [104,178,179]. The crucial issue of delivering mRNA into cells, as well as the critical contribution of formulation utilizing diverse delivery vehicles, must be given special consideration [80,180,181]. To safeguard our communities against worsening and future epidemics, high COVID-19 immunization rates are critical. Hundreds of millions of vaccination doses will require tremendous planning and implementation [182,183]. Despite the fact that this may be the world’s largest single vaccination attempt, best practices and lessons learned in pandemic preparedness, supply chain management, distribution, and clinical practice can help us immunize against SARS-CoV-2 [184,185,186]. To successfully manage vaccine delivery and administration to hundreds of millions of people, deliberate planning and coordination with local and international partners are essential [187,188,189]. Figure 3 shows the packaging and distribution process of the vaccines from production to use.

### 1.4. The Need for Novel Vaccine Delivery System

Despite the extreme discomfort involved with injections, expensive prices, and difficult injection schedules, many vaccinations and medicines need multi-bolus regimens, which can cause financial and emotional burden for patients [189,191,192]. Because of their restricted access to healthcare practitioners, people in underdeveloped nations face a greater challenge [77,163,193]. Low patient compliance, as well as other needle-and-syringe-related concerns, have been identified in several global health studies as major barriers to worldwide vaccination against deadly infectious illnesses including pneumococcal pneumonia [188,194]. The idea of a single-injection vaccine, which is acknowledged by the World Health Organization (WHO) as a preferred immunization method, has been studied for many years [195,196]. Furthermore, biohazards and the potential of disease transmission from the billions of needles/syringes discarded each year continue to be major problems with injection-based systems [155,197]. As a result, a novel medication and vaccine delivery strategy that is injection-free and only requires a single dose is urgently needed [198,199].

### 1.5. Microneedles in Transdermal Drug and Vaccine Delivery

Transdermal microneedles, which are painless and simple to use, have been shown to be an enhanced drug-delivery technique that allows for the less invasive administration of medicinal substances [159,200]. Transdermal microneedles are useful for vaccination because the presence of a significant number of immune cells (Langerhans cells) in the dermal layer of the skin improves immunogenicity [201,202]. However, microneedles only offer either rapid or sustained release, which limits their application in vaccine administration [203,204,205]. Due to the continual presence of the vaccine antigen inside the body, immediate-release versions may cause the formation of immunological tolerance against the vaccination, whereas sustained-release variants may induce the development of immune tolerance against the vaccine [206,207]. A transdermal microneedle device with programmable delayed burst release across prolonged time periods is required to replicate the traditional immunization process’s numerous bolus injections [208,209,210] This limitation is primarily due to a lack of a manufacturing technology capable of producing microneedles with core–shell or reservoir-based microstructures, which are required to provide pulsatile or delayed burst release with various desired lag times to mimic the drug-release pattern of multiple injections [10,211,212]. Recent advances in lithography-based methods and 3D printing have made it possible to construct drug-delivery devices with unique drug-release kinetics. Current lithography-based techniques can only generate two-dimensional structures, while current three-dimensional (3D) printing significantly relies on potentially hazardous impurities (such as ultraviolet-curing agents) [213,214,215]. Dissolving microneedles result in further advantages, both for the people who are vaccinated and for logistics, through providing tiny storage and disposal sizes, low-cost manufacture, and convenience of use, allowing for self-administration at home.

## 2. Dissolving Microneedles in Immunization

Dissolving microneedles (DMNs) are miniature needles made of polymers such as polylactic-co-glycolic acid (PLGA), polylactic acid (PLA), and polyglycolic acid (PGA) that dissolve in the skin to deliver encapsulated medicines, leaving no sharp waste [56,216]. DMN arrays are now overlaid onto patches to aid in their implantation into the skin [10,217]. The arrays produced on the patch are often not completely implanted, and significant amounts of loaded materials are not supplied due to substantial differences in skin elasticity and the amount of hair on the skin [213,218,219]. Drugs are most commonly delivered orally or by parenteral injection, among the numerous delivery methods available [60,61,220,221]. Thus, dissolving microneedles poses a suitable vaccine delivery system and is a worthy alternative for replacing traditional needle vaccination methods. The bioavailability of many orally administrable drugs is considerably decreased because of first-pass metabolism that can be affected by varied physiological elements such as the activity of enzymes, the level of serum protein, and the gastrointestinal motility of the drug in the body, although the oral dosage form is the most convenient method for drug administration [2,31,68,222]. For delivering medicines directly into the bloodstream, hypodermic injection is more convenient and can avoid the mentioned side effects [223,224,225]. However, there are several disadvantages to hypodermic injection, including the high degree of competence necessary to deliver an injection, the trypanophobia of some patients, and the danger of infection acquired by needle sticks on occasion [37,69]. Several researchers developed microneedle (MN)-mediated drug-delivery devices to overcome these restrictions, allowing patients to self-administer therapeutic micro- and macromolecule medicines without discomfort [226,227,228].

Dissolving microneedles (DMNs) are tiny needles made of polymeric materials that contain medications. The medication is released for systemic or local administration when DMNs are injected into the skin and catalyze the breakdown of the polymeric molecule [63,229,230]. DMNs are completely biocompatible and do not produce biohazardous sharp waste, unlike hypodermic injections [231,232]. Furthermore, as compared to subcutaneous vaccinations, DMNs have been found to be more dose-effective [233,234,235].

Currently, the only DMN application technique is to superimpose an array of microneedles onto patches, which allows for easier microneedle insertion and maintenance [41,236,237]. Patches are commonly employed as supports in DMN applications, although the efficacy of drug administration that may be obtained with patches is typically considerably decreased owing to excessive skin elasticity, which can result in inadequate DMN insertion [238,239]. Furthermore, the chemicals utilized in patch materials can cause skin irritation and/or allergic responses; other patch delivery drawbacks include difficulty sticking to flexible body joint regions and hairy skin [240,241,242]. Furthermore, before the patch can be removed, patients must wait for the DMNs to entirely disintegrate [222,243,244]. Table 5 shows the usefulness of dissolving microneedles in the vaccination procedures based on several criteria. Table 6 shows the different animal models for the use of dissolving microneedles for vaccination.

### 2.1. Fabrication of Dissolving Microneedles

Dissolving MNs are typically made by pouring liquid mixture into a previously prepared MN mold. In most cases, a silicon wafer is used as the starting material for the mold. The wafer is then oxidized at 1000 degrees Celsius. Lithography is utilized to create a needle geometry, followed by RIE, and CVD is used to coat a wafer. A liquid polymeric solution is put into the molds that have been produced. Air gaps are eliminated with a vacuum or centrifuge after a liquid polymeric solution is put into the prepared molds. After that, the mold is dried in an oven, and the MNs are removed after they have cooled. The advantages of this technique are that it produces MNs in a reasonably easy and cost-effective manner at room temperature.

The core–shell microstructure of the dissolving microneedles is created by assembling three separate components of the microneedles, including a microneedle shell, a microneedle cap, and a dry drug or vaccine core, utilizing a 3D manufacturing method [46,55,266]. The cap and base layer, which are composed of the same biodegradable polymer, poly(d,l-lactide-co-glycolide) (PLGA), encase the medication or vaccine core [212,216]. The drug release may be precisely regulated by adjusting the PLGA shell’s disintegration [217,219]. The microneedles may easily be implanted and thoroughly lodged into the dermal layer following fast skin healing due to their small points and smooth shape [267,268]. In theory, numerous sets of microneedles with various PLGA shells may be inserted into the skin of patients at the same time in the clinical environment to produce multiple burst release across different time periods, comparable to multiple bolus injections [61,269].

**Figure 4:**
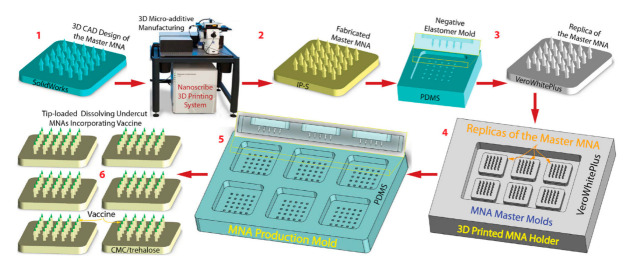
Dissolving microneedle array manufacturing strategy via micromolding (taken from [276]).

**Figure 5:**
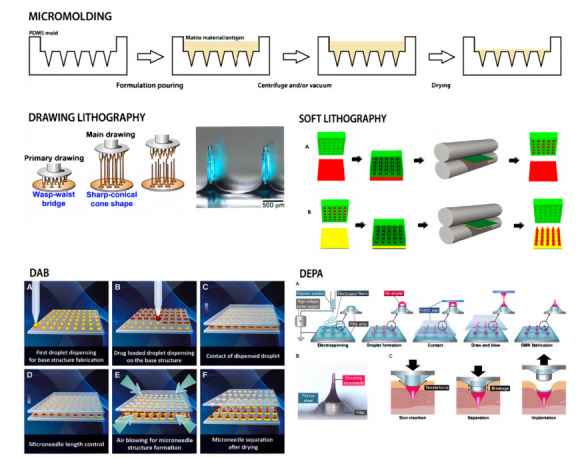
dMN manufacturing methods (taken from [277]) **Micromolding** with a polydimethylsiloxane (PDMS) mold is the most prevalent method for producing dMNs. **Drawing lithography** operates by extending two-dimensional polymeric material into a three-dimensional shape. Longitudinal extension of molten polymer by pillaring higher moving plate. In **soft lithography**, dMNs are produced by (A) heating a polymer sheet and a mold with microcavities. (B) The filled mold is then heated and placed on a flexible, water-soluble substrate. After mold detachment, a dMN patch remains on the substrate. **Droplet-born air blowing** (DAB) applies a (A) polymer solution and (B) a drug solution to two plates. (C) The upper plate is lowered until the droplets meet, (D)then withdrawn a distance equal to the two dMN lengths of the lower and top plates. (E) Drying the polymer solutions results in a dMN patch on each plate. (F) In addition, fabrication at moderate temperatures (4–25 degrees Celsius) minimizes medication and polymer waste. dMN on an **electrospun pillar array** (DEPA) is a variant of DAB. (A) The flat plate is replaced with a columnar array covered in a fibrous layer. (B) A PDMS slab is then utilized to draw and stretch polymer formulation droplets, resulting in microneedles. (C) Finally, the movement of air dries off the elongated droplets to form dissolving microneedles.

When different kinds of sugars are used as the matrix for dissolving microneedles, drugs or vaccines are usually released quickly in vivo [33,68]. For example, Ito et al. (2013) [254] reported that insulin was released from microneedles very quickly, with almost all the formulated insulin being released within 1 h when dextrin was used as the matrix [36,270]. Nonetheless, in some cases, a continuous release of medicines or vaccinations is necessary [271,272,273]. A prototype of DMNs was constructed by Lee et al. [274], in which therapeutic material was encapsulated merely as a backing layer that aided as a reservoir for the controlled release of therapeutic molecules by protruding it with interstitial fluid, which prolonged the molecule diffusion into the skin through the channels formed by DMNs [63,226,275]. Figure 4 shows the strategy of the fabrication of a dissolving microneedle array via micromolding, and Figure 5 shows different fabrication methods of dissolving microneedles.

The manufacturing approach for fabricating dissolving MNAs with new microneedle shapes is visually depicted in Figure 5. This six-step strategy takes advantage of AM and micromolding to generate dissolving undercut MNAs while also accomplishing high-throughput fabrication: (1) MNA design in 3D CAD; (2) the direct production of a master MNA from the CAD drawing by 3D direct laser writing using a non-dissolvable resin (IP-S); (3) the high-fidelity replication of master MNA by micromolding with UV-curable resin (VeroWhite); (4) the creation of MNA master molds consisting of multiple master MNA replicas on 3D-printed MNA holders; (5) the manufacturing of elastomer (PDMS) micromolding of MNA production molds; and the spin-casting of tip-loaded, dissolving MNAs with undercut microneedles containing a vaccine or other biocargo in a water-soluble biocompatible substance (e.g., carboxymethylcellulose (CMC) and trehalose). The final step of the process varies depending on the biocargo in question, but it usually involves spin-casting cargo (e.g., vaccine) into the tip of the PDMS production molds, followed by spin-casting a dissolvable hydrogel (e.g., CMC/trehalose) into the production molds to serve as the structural material.

**Figure 6:**
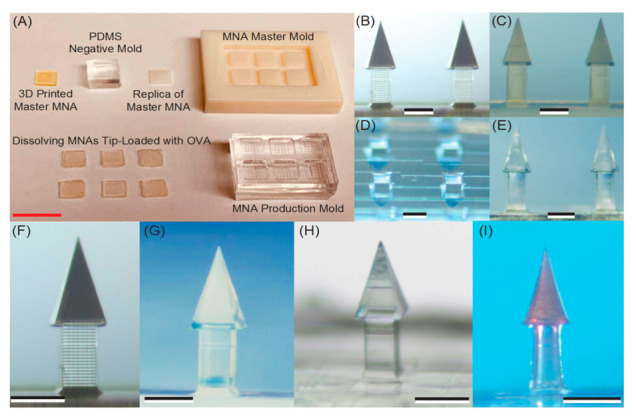
Fabrication of novel dissolving MNAs with undercut microneedles. (**A**) Finished items according to each stage of the production plan outlined. The scale bar measures 10 mm. (**B**–**I**). Optical stereomicroscopy was used to verify the geometric quality of the produced MNAs. The scale bars are 250 m in length. (**B**) Three-dimensional direct laser writing was used to generate the master MNA. (**C**) A two-stage micromolding approach was used to generate a replica of the master MNA (elastomer molding combined with UV-curable micromolding). (**D**) Wells formed like microneedles in an MNA manufacturing mold. (**E**) Dissolving CMC/trehalose MNA in the final stage, including a multicomponent vaccine (OVA + Poly(I:C)). (**F**) A closer look at a single undercut microneedle on the 3D-printed master MNA (as in B). (**G**) Magnification of an individual undercut microneedle on a master MNA replica at higher magnification (as in **C**). (**H**) Dissolving PVP/PVA microneedle tip filled with Alexa680-labeled OVA at the end. (**I**) A final dissolving CMC/trehalose microneedle tip filled with doxorubicin, a red-colored, chemotherapeutic, small-molecule medication, is shown. (Taken from [276]).

Biodegradable microneedles, which are made up of various biodegradable polymers such as polylactic acid, chitosan, polyglycolic acid, or poly(lactide-co-glycolide) (PLGA), break down in the skin after use, allowing the release of integrated medicines to be continued for months [230,231,232]. A recent study showed that these biodegradable microneedles might be used as a patient-friendly alternative to traditional sustained-delivery techniques [70,278,279]. However, to properly use the biodegradable polymer’s breakdown property, these microneedles must be implanted and left in the skin for several days [41,237,242]. The notable disadvantages of the fabrication of dissolving microneedles include manufacturing demanding the use of technical competence and time for the substance to dissolve. Microneedle separation into the skin was shown by Kim et al. [254] to be mediated by hydrogel swelling in reaction to contact with bodily fluid after the needles were introduced into the skin [243,280,281]. The hydrogel particles immediately absorbed water, causing the microneedles to break owing to the differential volume expansion between the needle–matrix polymer and the hydrogel particles [253,282,283]. The enlarged particles completely disintegrated the microneedles, leaving the microneedle tips in the skin of a porcine cadaver in vitro and a hairless mouse in vivo [284,285,286]. Figure 6 shows the fabrication of dissolving microneedle arrays with the PDMS micromolding technique.

### 2.2. Biodegradation Kinetics of Dissolvable Microneedles

**Figure 7:**
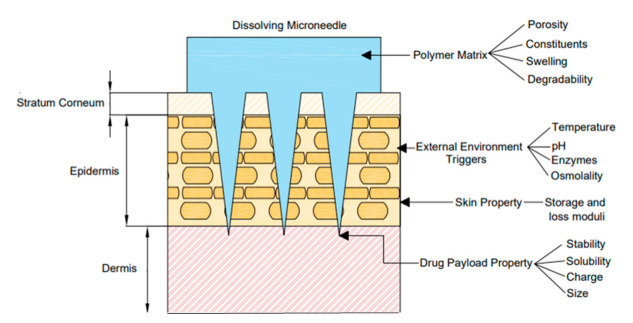
Schematic showing the variables which can affect the rate of drug release from a dissolvable microneedle (adapted from [289]).

Through considering the biodegradation kinetics of a DMN array as a mathematical model, the need for exploratory in vitro experiments during the design of new biodegradable matrix-based therapeutics can be reduced [287]. The development of such a model will allow key parameters such as DMN height, shape, and patch size to be optimized in a faster and more cost-effective way than by running laboratory experiments. However, the mechanism of degradation for a particular polymer is complex, as it depends on the properties of its polymer matrix such as its chemistry, molecular weight, and morphology [141]. It also depends on both the external environment, the payload property, and the skin property, as shown in Figure 7.

It is desirable to achieve a zero-order kinetics release profile during drug delivery to result in sustained release, which is independent of the concentration of the dissolved drug, and to prevent the drug concentration falling below minimum effective levels or rising above maximum safety levels [288].

However, this has been reported as idealized and difficult to achieve, with the majority of drug release profiles from polymeric drug-delivery systems resulting in a triphasic profile [289]. Although the release profile of each polymeric microneedle system should be evaluated on a case-by-case basis, some previous efforts to model degradation-controlled release, such as from a DMN, are outlined below.

### 2.3. Loading Capacity of Dissolvable Microneedles

Temperature-sensitive medicines such as peptides, antibiotics, and vaccines, as well as any temperature-labile treatment, may be bulk loaded into microneedle structures using room-temperature and aqueous-based micromolding [236,290]. Adjusting the postprocessing parameters of the microneedle structures, particularly the silk protein secondary structure, allows for the controlled release of a model drug [243,291].

**Figure 8:**
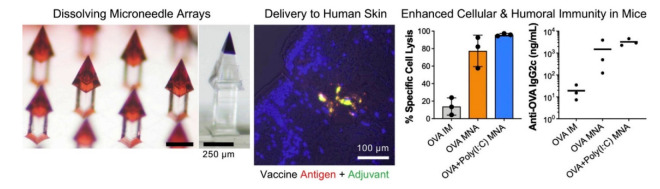
DMN vaccine delivery to human skin and induced immunity in mice (taken from [276]). *Note: OVA—ovalbumin (model antigen), IM—intramuscular, MNA—microneedle administration.*

Due to the bulk loading of dissolvable or biodegradable systems, microneedles efficiently bypass the epidermal barrier to provide this route as a viable option to the oral and parenteral administration of therapeutics, and relatively high dosages may be given [292,293] However, there are still a number of issues to be resolved. The skin’s barrier function, for example, varies from one location to the next on the same individual, from person to person, and with age [222,294,295]. Because the variation in individual skin and the penetration depth of microneedles are related to stress on the skin, an applicator may be necessary to produce a consistent penetration depth during each microneedle administration [63,296]. Furthermore, prolonged drug or vaccine release is more difficult than bolus release, and the kinetics should be researched and confirmed [232,297]. If microneedles are used often, it is also important to evaluate if dissolved or degraded matrices have any adverse effects [298,299,300] Microneedle vaccination elicited immune responses that were equivalent to those elicited by intramuscular injection in some cases but were greater in others [222,301,302]. Overall, microneedle immunization resulted in higher recall cellular immune responses, more antibody-secreting cells, and, most importantly, more effective viral clearance [33,70]. Figure 8 shows vaccine delivery into skin and induced immunity in mice.

### 2.4. Significance of Novel Transdermal Vaccination

Most vaccines are administered through injection, either intramuscularly or subcutaneously, which can be unpleasant and uncomfortable for individuals who are scared of needles [46,68]. Additionally, the hypodermic needles used to administer the vaccine in these methods generate hazardous waste, which might result in injuries and infection when needles are reused [243,290]. Infectious diseases such as Hepatitis B and AIDS can be transmitted through the latter, particularly in underdeveloped countries [46,55,266]. Moreover, the use of new vaccine delivery techniques may give a variety of other advantages, such as antigen thermal stability, fewer booster doses, and, as a consequence, greater vaccination adherence and a lower burden on healthcare personnel [236,239,290]. Both these advantages would be especially beneficial in large-scale vaccination operations, such as in the case of an outbreak, where feasible and rapid immunization procedures are necessary [56,63,249]. Because the skin is an immune-competent organ that is also easily accessible, dermal vaccination delivery seems intriguing [41,45,50]. In the viable epidermis and dermis, many antigen-presenting cells (APCs) such as Langerhans cells (LCs) and dermal dendritic cells may be detected (dDCs) [254,303,304]. Antigen-presenting cells gather antigens and subsequently transport them to draining lymph nodes, where they transfer the antigen to T cells, activating Ag-specific T cells and B cells for a systemic immune response [41,220,286]. Microneedles penetrate the skin barrier and underneath tissue to transfer the antigen into the epidermis or dermis while staying short enough to avoid pain receptors, therefore preventing pain sensation [261,305]. Additionally, MN vaccination may not require the employment of health practitioners and will not result in sharp needle waste after immunization [37,53,305,306].

### 2.5. Mathematical Modeling of Microneedles

**Figure 9:**
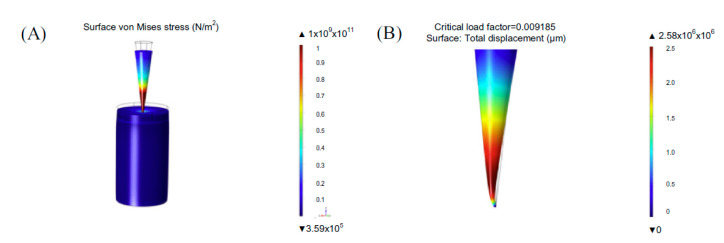
Finite element analysis of a CMC/maltose microneedle with a conical shape. (**A**) Surface von Mises stress when an axial load of 5 N is applied on the base. (**B**) Prediction of bucking mode when an axial load of 5 N is applied at the base and a fixed constraint is forced at the tip [306].

The mathematical modeling of microneedles for vaccine delivery is key in optimizing the MN performance. Currently, there are few publications involving the modeling and optimization of DMN arrays when compared with solid or hollow microneedles. Consequently, this section aims to summarize the drug-delivery mechanisms discussed within these papers along with the key parameters that have been found to affect the drug release rate from the microneedle array and the microneedle penetration depth with a view to learn the key lessons from these publications. To date, the method chosen by most studies to model DMNs was the finite element method (FEM) [158]. This is because this method is able to produce detailed diffusion or force distribution profiles [304,306,307]. Figure 9 shows a finite element analysis of the surface von Mises stress on a single microneedle.

#### 2.5.1. MN Delivery Mechanisms

This section discusses the fundamental principles used to derive the DMN mathematical models reported within the literature to date. To successfully deliver a drug, a DMN system must be applied to the skin to puncture the stratum corneum and permeate the upper dermal layers. Once the needles are “wetted” by moisture in the skin, they will dissolve to release drug molecules which are eventually adsorbed into the circulation [307]. Typically, Fick’s law can be used to simulate the diffusion profile of the drug molecules in microneedle-treated skin. Fick’s first law is used to describe steady state diffusion. Fick’s second law is used to describe transient diffusion [158].

The value of diffusion coefficient used in Fick’s law can be calculated using various methods, described in detail by Yadav et al. [141]. Chavoshi et al. [308] also reported that the drug diffusion coefficient will increase as polymer degradation occurs. Despite this, many models of DMNs assume that the drug concentration in the skin layer is uniform because the rate of diffusion is rapid compared to the dissolution of the microneedle or elimination into the bloodstream [309,310].

Ronnander et al. [309] developed a mathematical model to show the in vitro dissolution and release of sumatriptan succinate from PVP-based microneedles shaped as pyramids. To do this, governing equations were derived using material balances to relate the microneedle pyramid height and drug concentration in the skin over time.

Kim et al. [310] used a similar approach to predict the amount of drug (fentanyl) delivered into the skin via the dissolution of a water-soluble (sucrose) microneedle. More recently, this approach was used by Zoudani and Soltani [311] to create a numerical simulation of a dissolution process of a DMN in porous medium. However, Kim et al. [310]; Ronnander et al. [307]; Zoudani and Soltani [311] assumed that the ratio of needle height to the base radius remains constant throughout the dissolution process. This may not always be true; therefore, a more accurate model would consider these variables independently.

When creating their numerical simulations, Zoudani and Soltani [311] introduced a hindrance factor to investigate the effect of a drug’s molecular radius on a drug’s effective diffusion coefficient in the skin. This was not considered in the studies completed by Kim et al. [310] or Ronnander et al. [307].

#### 2.5.2. Effect of Polymer Type

To date, there have been various studies completed to optimize the polymer used in a DMN array [306,309,312,313,314]. Each study has been undertaken with the aim of providing a formulation with optimum values for Young’s modulus, Poisson’s ratio, ultimate tensile stress, dissolution kinetics, and polymer adsorption rate. Moreover, there are a good number of articles (e.g., [47,222,315,316,317]) that discuss the varied types of materials usually used to fabricate MNs.

To study the traits of sugar MNs, Loizidou et al. [306] used finite element analyses which was the first initiative undertaken with the objective of optimizing the polymer. They evaluated the effects of sugar composition on MNs’ capability to penetrate and dispatch therapeutic materials through the skin. MNs made from CMC/maltose were found to be better than those made from CMC/trehlose and CMC/sucrose in terms of their mechanical strength and ability to deliver drugs. Loizidou et al. [306] also stated that the main mode of microneedle failure is buckling, which is positively correlated to the Young’s modulus of the microneedle array.

Amodwala et al. [318] completed a similar study to optimize the ratios of PVA to PVP and the solid content of a matrix to achieve maximum microneedle strength. The optimum patch was found to contain a 9:1 PVA to PVP ratio with 50% solid content. This formulation showed a maximum needle fracture force of 0.9N and was found to release 100% of the encapsulated drug (meloxicam) in 60 min [312]. Similarly, Ronnander et al. [309] looked at different ratios of water, sumatriptan succinate, and PVP within a DMN array and found that the formulation affects the drug release rate and time needed for the polymeric microneedle to dissolve.

Suriyaamporn et al. [314] used computer-aided rational design to optimize the formulation of Gantrez- and hyaluronic-acid-based DMNs as a potential ocular delivery system. The optimal DMN formulation was found to be 20.06% Gantrez +5% hyaluronic acid +1% Fluorescein Sodium, as it gave the optimum combination of dissolution time, insertion force, and insertion depth.

However, the simulations of drug delivery using DMN can be advanced through looking at the interactions between the polymer and drug in the microneedle structure. Hao Feng et al. [313] used molecular dynamic simulations to model the binding energy and electronegativity differences between polymer and drug molecules. This study is essential for determining compatibility between the polymer and loaded drug, therefore allowing for efficient drug delivery and minimal wastage of drugs.

#### 2.5.3. Effect of Microneedle Array Geometric Parameters

In a microneedle array, properties such as the needle length, tip radius, base diameter, center-to-center spacing between two microneedles, the number of microneedles, and the distribution of microneedles in an array work together as a synergetic system. The needle geometry, thickness, and density are also parameters which will affect the concentration of active pharmaceutical ingredients in the blood [157].

Various publications have shown that increasing the pitch width between microneedles in an array will reduce the level of drug in the dermal layer of skin [311,312]. However, it has been further suggested that the effect of microneedle pitch on skin permeation is non-linear, and decreasing the pitch size has no significant effect on dissolution time [309]. Despite these findings, during their study on the amount of drug delivered into the skin via the dissolution of a water-soluble microneedle, Kim et al. [310] neglected the effect of the pitch and needle geometry on the deformation of skin. Therefore, these effects need to be considered to increase the accuracy of this model.

Chen et al. [319] found that polymeric microneedles with a longer length presented higher TDD efficiency. The drug used in this study was insulin, and the microneedle lengths varied between 124 µm and 445 µm. These results can be explained by considering the microneedle volume present in the viable epidermis and dermis layers of skin, which have increased transport properties when compared to the stratum corneum [320]. As the needle length increased, the percentage of needle volume present in the viable epidermis and dermis increased, leading to an increased release of insulin. Despite these findings, the author noted that using longer polymeric microneedles may not be optimal, as shorter lengths would significantly reduce the pain due to skin piercing. Gomaa et al. [321] also found that longer microneedles may require a greater insertion force for their use to be effective.

**Figure 10:**
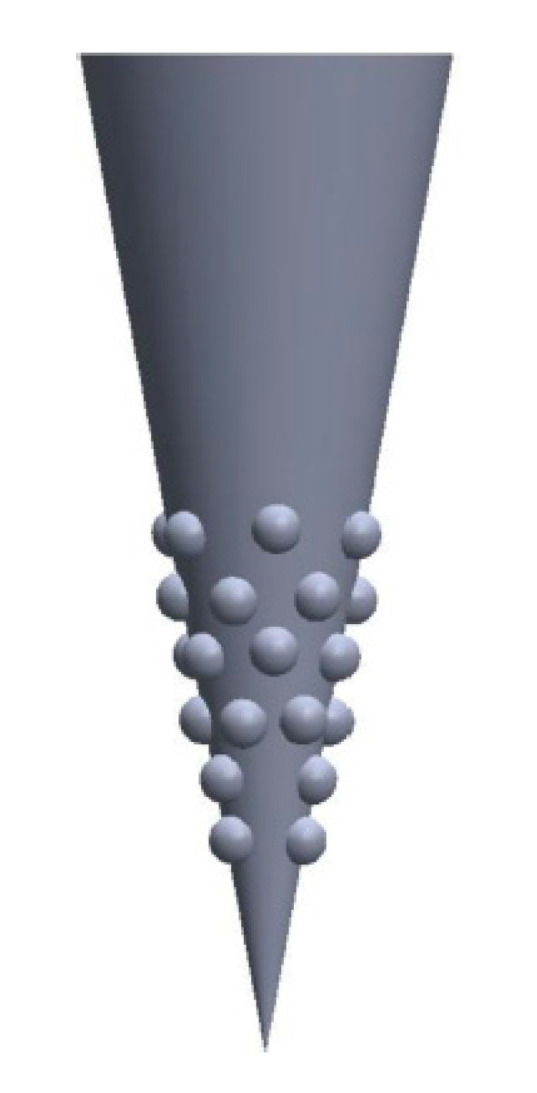
Configurations of the microneedle shape [311].

Zoudani and Soltani [311] proposed a new approach called array in array theory, a cone with an array of hemispherical convexities located in the second half of the microneedle, as shown in Figure 10. According to the numerical simulation of this design, the drug concentration left in the tissue was double the concentration left from a conical design; however, the time taken for the needle to be fully dissolved was unaffected. Therefore, the new configuration led to a more effective and economic method of drug delivery through a DMN array [322]. Despite this, a much more complicated fabrication procedure is expected for such devices.

#### 2.5.4. Effect of Skin Properties

Skin has a viscoelastic property, which must be considered when modeling the insertion behavior of a DMN array [158]. Skin thickness, Young’s modulus, porosity, and viscoelasticity are important parameters that may affect the DMN penetration depth.

As aforementioned, Loizidou et al. [306] performed experimental and finite element analyses to study the mechanical properties of sugar microneedles when inserted into skin, as shown in Figure 10. However, there are fewer publications on the insertion behavior of DMN compared to solid or hollow MNs. Although polymeric microneedles are soft compared to solid microneedles, the principles of microneedle insertion into skin remain the same.

## 3. Dissolving Microneedles: Some Satisfactory Aspects

**Figure 11:**
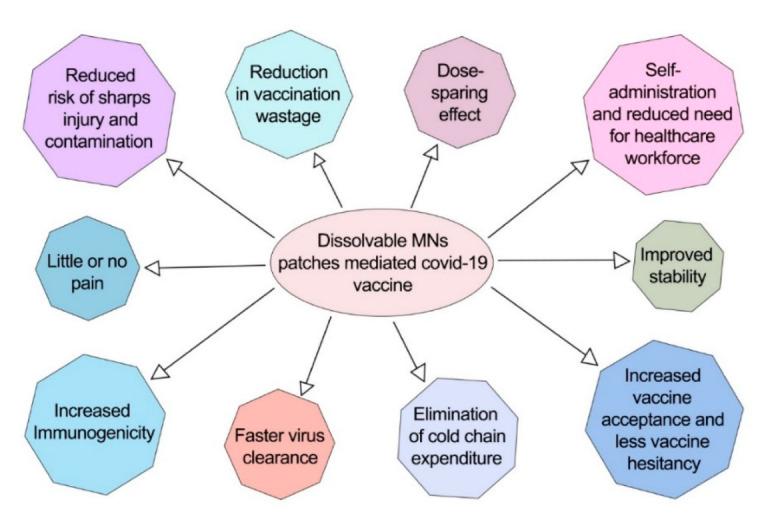
Advantages of dissolvable MN-mediated COVID-19 vaccine (information taken from [329]).

Transdermal medication has become a very popular, effective, and promising administration route for drug delivery, and the concept of microneedles has intensified this [55,323]. Researchers have successfully established vaccination delivery via MNs through numerous studies [39,261,265,324,325,326,327,328], and now, DMNs have attracted immense attention for COVID-19 vaccine delivery. Researchers are optimistic about using DMNs for mass vaccination for COVID-19 due to the reasons outlined in Figure 11.

Research works that support the dissolvable-MN-patch-mediated COVID-19 vaccination system are displayed in Table 7.

### 3.1. Patient Compliance

MNs are safe therapeutic devices that do not require highly experienced or properly trained caregivers to administer them [360]. MN patches can be used to self-administer vaccinations [59,246]. MNs are the best possible suitable vaccination option for people with needle phobias [54]. In addition, MNs do not cause irritation after administration and are less painful than conventional syringes [34,42,298,361,362,363,364]. DMNs are the best possible substitute and hold the potential to be better than conventional syringes [346] because of the following reasons:

### 3.2. Overall Vaccination Cost Reduction

As DMNs are fabricated from dissolvable polymers, they have the potential to eliminate the manufacturing cost of syringe and vials [163,170,329,365]. DMNs also have the potential to reduce the storage, distribution, and overall manufacturing cost of COVID-19 vaccinations. Table 8 summarizes how DMNs will reduce the total cost of COVID-19 mass vaccination.

## 4. Preclinical and Stability Studies of MN Vaccination

### 4.1. Stability Studies of MN Vaccination

Most immunizations are currently accessible in fluid form, which must be kept refrigerated to guarantee antibody quality [342,352]. Due to this tight temperature prerequisite, immunization administration by and large utilizes a cold chain, which may be a set of temperature limitations that happen amid immunization travel, capacity, and dissemination from the point of fabricating to the point of utilization [12,87,366]. Indeed, a well-established cold chain, be that as it may, cannot guarantee immunization quality, since any accidental introduction to warmth or the unintended solidifying of immunizations amid travel and capacity can cause damage [239,249,277,361]. Due to the colossal cost of keeping up the cold chain and adapting to its issues, antibodies with thermostability that do not require refrigeration are significantly sought after [49,63,345]. Thermally steady immunizations could be distributed to populaces in nations with restricted cold-chain frameworks, and more productive and far-reaching immunization dissemination could be achieved through drugstore and mail-in techniques to combat regular and widespread episodes of maladies such as COVID-19 [233,344,355]. Table 9 shows stability studies regarding dissolving microneedles used in vaccination. Changing fluid antibodies to a dry powder frame is one approach to improve immunization toughness [232,341,350]. In terms of drying, vacuum drying, drying with a desiccant, and lyophilization are all methods of drying biopharmaceuticals such as immunizations [78,100,102]. The prescribed procedure for drying biopharmaceuticals is lyophilization [33,40,339,367]. Amid the freeze-drying process, be that as it may, the immunization particles and proteins are exposed to a number of possibly damaging stresses, including solidifying and drying stresses, which can cause changes within the antibody proteins’ auxiliary and tertiary structure as well as physical changes such as conglomeration (e.g., due to solidifying concentrations) [33,46,55,236,332]. Sugar has been utilized in studies regarding antibodies to protect immunizations against the previously mentioned types of damage during lyophilization, as often as possible within the frame of sugar glass [41,284,368,369]. In spite of the fact that the most popular way of conveying immunizations is infusion with hypodermic needles, this strategy is not favorable and our understanding of it is limited [37,67,370]. In numerous developing nations, it was assessed that more than half of all infusions are performed utilizing dangerous infusion strategies, which may be a major source of bloodborne pathogen transmission [338,368]. Microneedles, alternatives to infusion, tackle these concerns by giving a more patient-friendly and more secure conveyance strategy that infuses immunizations into the epidermis and shallow dermis layers of the skin, utilizing an easy-to-apply alternative [63,156,239,332,345]. Patients lean toward the microneedle alternative since it is easy and simple to apply, and it is more secure, since microneedles may be fabricated from secure, water-soluble excipients that break down within the skin and leave no sharp waste [46,49,55,371]. In addition, microneedle patches can be stored and dispersed in a dry, solid-state and are broken down within the skin’s interstitial liquid when utilized [63,77,345]. Further examinations have found that skin immunization is more immunogenic than muscle immunization, owing to the nearness of Langerhans and dermal dendritic cells within the skin [233,341,344]

### 4.2. MN Patch Packaging and Storage

In a previous study [352], three alternative packing conditions were used to preserve microneedle patches carrying vaccinations [195,357,373]. The initial set of microneedle patches were put in open glass vials that were exposed to the building’s ambient air and humidity [239,351,374]. The remaining microneedle patches were put in glass vials with 1 g of desiccant (calcium sulfate, Drierite, Xenia, OH). These vials were securely capped and then parafilm-sealed. The third batch of microneedle patches was likewise packed in glass vials with desiccant and nitrogen gas instead of air [55,156,233]. Microneedle patches were kept at 4 °C in the fridge, 25 °C on a lab bench drawer, and 37 °C or 45 °C in temperature-controlled incubators [33,40,342]. At the four temperatures, fluid arrangements of inactivated flu infection in vials were kept indistinguishably with and without desiccant and oxygen [33,249,277,284,355]. After 0, 1, 7, 14, 30, 60, and 90 days, the microneedle patches and immunization arrangements were expelled, and their solidness was tested [163,355,369,370]. Table 10 shows the analytical methods used to monitor and determine the quality attributes and to test the quality attributes of mRNA vaccines.

### 4.3. Preclinical Studies of Vaccine MN Array Patch

Mice were utilized in the study conducted in the article [352] to assess the viability of most immunization MAPs, whereas a monkey was utilized as an animal model in preclinical examinations of distinctive immunization MAPs [63,370]. An aluminum-type adjuvant was tested among the adjuvants; in any case, it did not illustrate high proficiency for the MAP14 immunization since it produced destitute T-cell interceded resistant reactions and was not fitting for intradermal (ID) utilization [50,63,74,202]. Nanoparticles (NPs), which can work as a station and are more effectively taken up by dendritic cells, have as of late been respected as valuable adjuvants. Bacillus anthracis inoculation NP Outlines evoked a more capable resistant reaction than an Outline without NP detailing [50,63,144,200]. Ebola immunization studies indicated a comparable advancement [63,376,377]. The Hepatitis B DNA antibody was typified in another polymer NP detailing made from pluronic-modified polyethyleneimine [63,370]. Compared to DNA MAPs, DNA NP MAPs created more prominent humoral and cellular resistance [50,63,74,202]. Utilizing an embedded Outline, the resistant reaction was upgraded by a persistent discharge of inoculation and antigen presentation to lymphoid organs [140,145,325,377]. The silk network was utilized to form a D-MAP for HIV that controlled the antigen discharge rate for two weeks, occurring in a 1300-fold increment in serum IgG titer compared to a conventional organization [33,203,303,368]. The chitosan Outline, which has an immune-enhancing impact, was utilized to extend the discharge of flu inoculation [33,97,140,156,200,325,376].

## 5. Microneedle Array Patch Vaccination: Clinical Trials and Human Studies

### 5.1. Microneedle Vaccination Clinical Trials

To address the limits and present drawbacks of hypodermic needle injection, vaccines can incorporate MAP’s innovation in stability, bioavailability, potency, and less adverse effects [74,150,208]. Using the keyword “microneedle vaccination” as a search term, seven studies were discovered on Clinical Trial.gov that used the vaccine MAP. In the registered trials, many types of MAPs were employed, and these studies were performed to assess the viability of MAP vaccination in clinical practice for some of the most severe infectious illnesses [22,68,83,208].

### 5.2. Vaccine Coated MN Array for Human Studies

In multiple human trials, C-MAP (NanopatchTM), a vaccine, has shown promise as a technique for effective drug delivery [63]. In a previous study [63], uncoated and excipient-coated NanopatchTM vaccines were given to 18 healthy persons for 2 min of insertion and removal [33,368,370]. On a scale of 0 to 10, 78 percent of participants reported 0 on a pain range of 0 to 10, with an average score of less than 1 on a pain scale of 0 to 10 [58,59,60,61,62,63,64,65,66,67,68,69,70,71,72,73,74,75,76,77,78,79,80,81,82,83,84,85,86,87,88,89,90,91,92,93,94,95,96,97,98,99,100,101,102,103,104,105,106,107,108,109,110,111,112,113,114,115,116,117,118,119,120,121,122,123,124,125,126,127,128,129,130,131,132,133,134,135,136,137,138,139,140,141,142,143,144,145,146,147,148,149,150,151,152,153,154,155,156,157,158,159,160,161,162,163,164,165,166,167,168,169,170,171,172,173,174,175,176,177,178,179,180,181,182,183,184,185,186,187,188,189,190,191,192,193,194,195,196,197,198,199,200,201,202,203,204,205,206,207,208,209,210,211,212,213,214,215,216,217,218,219,220,221,222,223,224,225,226,227,228,229,230,231,232,233,234,235,236,237,238,239,240,241,242,243,244,245,246,247,248,249,250,251,252,253,254,255,256,257,258,259,260,261,262,263,264,265,266,267,268,269,270,271,272,273,274,275,276,277,278,279,280,281,282,283,284,285,286,287,288,289,290,291,292,293,294,295,296,297,298,299,300,301,302,303,304,305,306,307,308,309,310,311,312,313,314,315,316,317,318,319,320,321,322,323,324,325,326,327,328,329,330,331,332,333,334,335,336,337,338,339,340,341,342,343,344,345,346,347,348,349,350,351,352,353,354,355,356,357,358,359,360,361,362,363,364,365,366,367,368,369,370,371,372,373]. NanopatchTM had no unexpected adverse effects, and the expected erythema response faded between three and seven days after vaccination [56,61,249]. When healthy people were administered a NanopatchTM containing 15 g of inactivated influenza virus (H1N1), the side effects were low to moderate, and more than half of the people (55%) preferred the NanopatchTM method over intramuscular injection (IM) [58,209,249]. Interestingly, when employing a NanopatchTM, the antibody response was comparable to when using standard IM injection [33,56,61,368,370,373].

### 5.3. Vaccine Dissolvable MAP for Human Studies

There was no pain, edema, or erythema when research participants in a number of studies [63,326,368] were administered a D-MAP patch, and only mild erythema was restricted to the patch application site [35,326]. Furthermore, the great majority of participants were either somewhat or totally confident in their ability to self-administer [35,50,290,326]. As a consequence, D-MAPs were given to participants in a phase 1 research after the influenza vaccine was encapsulated in a polymer matrix [33,35,38,340]. The D-MAP that was self-administered generated antibody responses that were comparable to IM therapy [51,260,354]. Another D-MAP for the treatment of influenza was created using MicroHyala TM, a hyaluronic acid MAP [63,64]. There were hardly any significant local or systemic adverse effects, and the immunological efficacy was comparable to IM [41,43,284].

### 5.4. Concerns about Vaccination via MAP

#### 5.4.1. Commercialized MAP

A few pharmaceutical companies have developed MAP devices for medication delivery systems. These companies are summarized in Table 11. OnvaxTM (a BD business) is made up of a series of plastic microprojections that stand about 200 micrometers tall [222,353]. The vaccine is transported into the epidermal layer as a result of the skin injury induced by such devices [33,43,63,326]. Vaxxas created NanopatchTM, which is a strong power microprojection grid with an influenza vaccine composition on top [50,222,353]. A spring-loaded applicator is used to apply a 250 m long NanopatchTM needle. The ZP MAP system from Zosano Pharma contains 1300 microneedles distributed out across a 2 cm2 area [36,46,63,232]. The medicine is deposited on a 190 m MAP and given through a refillable arm applicator. This technique accomplishes the intended outcome by delivering the drug formulation to the skin’s outer layers. CosMED Pharmaceutical Ltd. created a D-MAP that is built on hyaluronic acid [222,353].

#### 5.4.2. Manufacturing Issues

Dosage consistency, reasonable price, mass fabricating, and manufacturing in agreement with GMP benchmarks are all concerns in attaining successful DMN vaccination procedures [44,55,222]. In terms of cost and versatility, MAPs require the foundation of large-scale fabricating apparatus and methods [43,58,276,324]. At the beginning of mass generation, noteworthy use is required to manufacture, cast, and shape MAPs [32,68]. Once these early stages are complete, the fabrication expenses of DMNs are anticipated to be lower than that of injectables [32,33,37,46,50]. The polymer-based MAP casting technique might be low-cost since different polymers, such as cellulose derivatives, engineering plastics, and sugars, are typically affordable [41,46,66,304,369]. Scaling up handling processes, however, can be challenging due to the reliance on master molds and the necessary multi-step filling process [50,56,69,225]. Additionally, temperatures may rise due to the drying process utilized in the manufacturing of vaccine MAP [63,254,378,379]. As a result, making thermosensitive antigens may need a low-temperature technique. Special packaging or desiccants may be required to improve storage stability, although the addition of moisture-resistant material may increase package prices [36,233,236]. In addition, sterilization is essential for MAP immunization. Despite MAP’s low bioburden, the cost of the aseptic technique should be considered. The assessment of immunization MAP products should be considered in terms of cost [33,251,326]. The consistency of MAPs is also necessary for quality assurance in the marketing and production processes. Companies must establish and execute an effective pharmaceutical quality assurance system that combines GMPs and quality risk management [63,145,249]. Ultimately, for effective vaccine MAP production, current standards for conventional medications, as well as specific needs for each type of MAP, are necessary [33,63,326].

#### 5.4.3. Regulatory Issues

An immunization Outline, agreeing to the latest report from the US Food and Drug Administration (FDA), could be an item that combines an organic item with a mechanical device [78,116,376,377]. Two or more administrative units such as the organic item and the mechanical device are combined to make up an item. This sort of item incorporates prefilled syringes, autoinjectors, and Outline patches preloaded with natural items (21 CFR 3.2e) [9,156,158,175,305]. Due to the benefits of Outline, an inoculation Outline has been focused on. Antibody and Outline were combined into one commerce [88,305,372]. The security and viability concerns related to each constituent portion and the item as an entirety ought to be considered within the direction of an immunization Outline (21 CFR Portion 4 Subpart A: Section 4.4 (b)). To move from the research facility to clinical utilization, an immunization Outline must moreover meet current GMP and post-marketing security benchmarks [61,88,163,305,370,378].

## 6. Challenges in Ensuring Global Access to COVID-19 Vaccines and Socio-Economic Factors

### 6.1. Vaccine Hesitancy

Vaccine hesitancy is defined as the delay in receiving or the refusal to receive vaccines in spite of the availability of a vaccine facility [379]. The severity of the COVID-19 pandemic cannot be minimized until communities agree to get vaccinated [380]. According to some researchers, people who prefer to receive complementary and alternative medicines (CAMs) are more prone to become vaccine hesitant [381].

In this ongoing pandemic, one of the reasons people are showing hesitancy towards the vaccine is that the COVID-19 vaccine development time period was faster than usual [153]. This has led to a fearful doubt regarding the long-term effect of the vaccine [82].

Despite this, reasons for vaccine hesitancy may vary according to country, socio-economic factors, one’s confidence towards the vaccine, and other factors [152,382]. Moreover, a lack of proper vaccination campaigning, ignorance of vaccination, and media communication have a substantial effects on people [148,205,383,384,385]. Numerous surveys have been carried out to identify the exact reason for vaccine hesitancy, and each of them included a considerable number of participants who lacked knowledge concerning the COVID-19 vaccine [5,81,89,386,387]. Media coverage regarding the adverse effect of AstraZeneca’s vaccine also led many people towards vaccine hesitancy [116,385].

To tackle the growing percentage of vaccine hesitancy during the COVID-19 pandemic, the first and foremost step is to increase awareness among the public. This involves disseminating information, educating people about the importance of vaccines, and fighting disinformation with scientific-data-based information [130,148].

Researchers have suggested the five Cs strategy to combat vaccine hesitancy, which covers: 1. Confidence, 2. Complacency, 3. Convenience, 4. Communications, and 5. Context [151].

**Figure 12:**
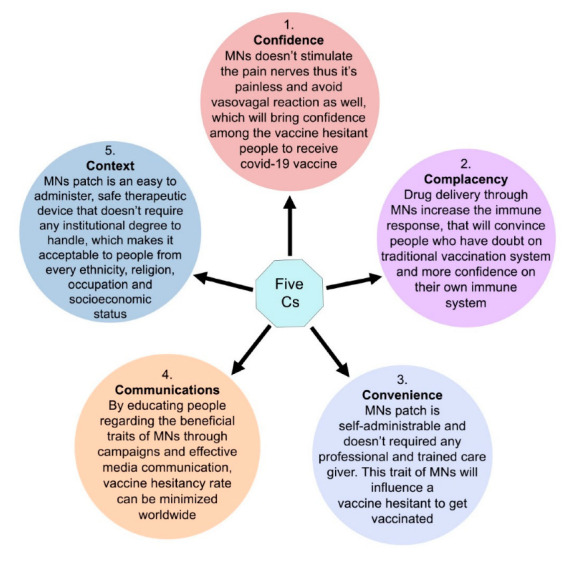
Role of MNs in dealing with vaccine hesitancy.

MNs can play a vital role in reducing the percentage of vaccine-hesitant people worldwide. Because it is now proven that delivery through MNs can increase the effectiveness of a medication, not only that will lead to confidence in vaccine-hesitant people, but it also has the potential to deal with the other four components of the five Cs (see Figure 12).

### 6.2. Needle Phobia

A needle phobia can be defined as the intense fear of medical approaches that involve needles or injections, to such extent where it causes a transformed and unaccommodating response [388]. The intensity of this phobia can reach to an extent where a patient might even refuse to accept life-saving medical help [389].

A needle-phobic human usually goes through a series of phenomena, initially starting with anxiety-associated tachycardia, and subsequently bradycardia, hypotension, diaphoresis, and shock. Eventually, all these incidents can lead a needle-phobic towards vasovagal syncope. Unfortunately, this fear of vasovagal syncope leads a treatment receiver to experience more severe needle-phobic responses than usual [389,390,391]. All these medical conditions discourage people with needle phobias to get vaccinated despite the long-term beneficial effect of vaccines.

To convince people with needle phobias to get vaccinated, the following methods could be applied:

1. Desensitization: Desensitization therapy is the most commonly applied and effective; however, it is a time-consuming technique that requires hours of compliance. In this therapy, the patient is steadily exposed to needles in a regulated and circumspect setting that eventually helps the needle-phobic to allow themselves to handle needles [390,392].

2. Topical anesthetics: The use of topical anesthetics before the administration of transdermal medications is the best possible method in the management of needle phobias, which can temporarily solve the associated issues within the shortest possible time. Local anesthetics that are cream-based mixtures have shown more efficacy in the management of needle phobias [389,393].

3. Vaccination through MNs: MNs have become popular therapeutic devices [36] for vaccine delivery due to their distinctively unique advantages [14], which can play a paramount part in alleviating the fear of needles in people. MNs are tiny, micron-sized needles that deliver the drug molecule by creating microchannels through the stratum corneum [46,364] without stimulating pain nerves [360,394]. They can penetrate the skin without pain and vasovagal reactions, making them a suitable transdermal delivery system for needle-phobic people [31,47,395].

### 6.3. Availability

**Figure 13:**
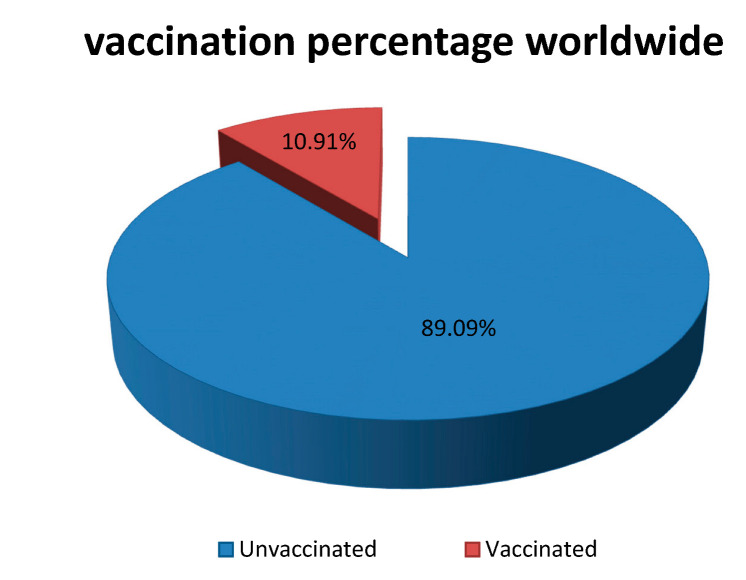
Percentage of unvaccinated people worldwide as of 1 June 2021.

As it has already been almost 2 years since the beginning of the COVID-19 pandemic and only 10.91% of people have been fully vaccinated as of 1 June 2021 [396], it is a challenge to fully vaccinate the rest of the 89.09% unvaccinated people (Figure 13) worldwide in spite of the remarkably rapid development of COVID-19 vaccines.

Based on the report of an internal investigation, the amount of vaccines supplied altogether by 37 members of the DCVMN (Developing Countries Vaccine Manufacturers Network) was about 3.5 billion doses annually [95].

According to the latest published article on 9 June, around 20 million people per day had been getting a vaccine shot manufactured by Sinopharm and Sinovac in China [397].

**Figure 14:**
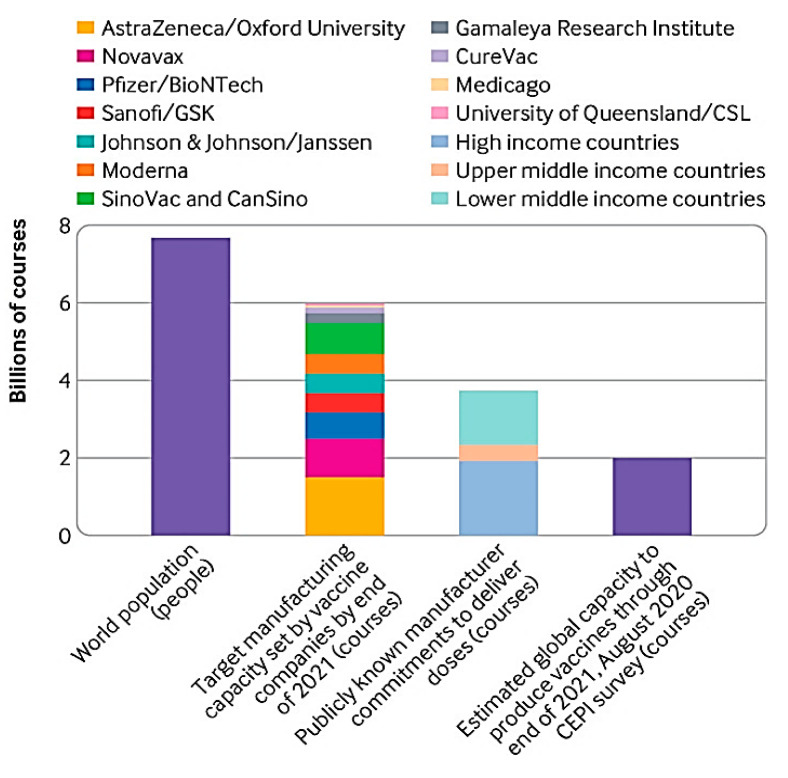
Manufacturing capacity by end of 2021 of lead companies producing COVID-19 vaccines. (Vaccine courses are assumed to require two doses) [399].

AstraZeneca agreed to deliver 3 billion doses of its vaccine this year; however, it was only able to deliver 40 million of the 90 million doses it had promised to provide the European Union (EU) for the first quarter of 2021 [398]. Figure 14 depicts a general estimation of the COVID-19 vaccine manufacturing capacity of leading manufacturers worldwide by the end of 2021.

The COVID-19 pandemic has given rise to a humanitarian crisis throughout the world. Developing and underdeveloped countries have always been at the bottom of the priority list in the case of both technological and medical advancements, which also include vaccines and drugs [130]. For instance, the world’s poorest nations in Africa only received 0.2% of the 700 million COVID-19 vaccine doses by April 2021, whereas more than 87% of the global vaccine doses has been supplied to developed countries [176].

However, a variety of agreements with manufacturers has been launched by the COVAX Facility regarding the supply of COVID-19 vaccines, which it believes will be sufficient enough to hit its target of producing two billion doses by the end of 2021, around 50 percent of which will be reserved for distribution in developing countries [400]. Canada, France, Norway, and the UK have agreed to donate leftover vaccines from their national vaccination campaigns [398].

The pervasiveness of vaccine nationalism is a barrier to equitable vaccine distribution [400].

In an endeavor to reconcile the different perspectives, some people think that no conflict exists in terms of an even distribution of vaccines and national partiality. Having said that, it is not allowed to violate civil rights and aggravate the worldwide poverty situation. Furthermore, it is both the right and duty of each government to ensure their citizens get priority access to the COVID-19 vaccine [127].

#### Available Vaccines and Variants of Concern

SARS-CoV-2 is susceptible to genetic evolution that results in multiple variants that possibly have distinct characteristics in comparison to its hereditary strains. In the past year, multiple variants of SARS-CoV-2 have been originated, of which a few are considered to be a variants of concern (VOCs) [401] because of their potential to cause enhanced virulence, reduced naturalizing capacity by the help of innate immunity or vaccination, the ability to elude the detection, or the decreased effectiveness of therapeutics or vaccination [136]. Each EUA vaccine available on the market evinces a different potency and time span of effectiveness depending on the design of antigen, adjuvant molecules, vaccine delivery platforms, and immunization technique [114]. The efficacy of EUA vaccines present on the market for the different variants is as follows:

**BNT162b2 vaccine:** The roughly calculated effectiveness of the BNT162b2 vaccine was 89.5% (95% confidence interval (CI), 85.9 to 92.3) and 75.0% (95% CI, 70.5 to 78.9), against infection with the B.1.1.7 and B.1.351 variant, respectively, at the 14th or more day after the administration of the second dose [84,101,103,402,403,404]. BNT162b2 efficiently neutralized all SARS-CoV-2 variants in the in vitro analysis of 20 serum samples attained from 15 participants from the BNT162b2 clinical efficacy trial. The neutralization of the B.1.1.7 variant and P.1 was unceremoniously identical. The neutralization of B.1.351 was stronger but lower compared to the hereditary strain of SARS-CoV-2 [109,401,405].

**Ad26.COV2.S vaccine:** A single course of the vaccine offers protection from the P.2 and B.1.135 variants of COVID-19, though data have not been reported yet. It is notable that the efficacy [406] of the vaccine in the US was higher by a factor of 1.3 in comparison to South Africa, which was 72% and 57%, respectively [407].

**mRNA-1273 vaccine:** The percentage of effectiveness of the mRNA-1273 vaccine against the SARS-CoV-2 variants is not clear yet [27,167,169,408]. The in vitro experiment of serum samples collected from members who participated in the mRNA-1273 vaccine’s clinical efficacy trial shows that the mutations affecting the receptor-binding domain (RBD) of the B.1.1.7 strain had no remarkable effect on neutralization by serum collected from participants who received the mRNA-1273 vaccine. On the contrary, the analysis also demonstrated a reduction in titers of neutralizing antibodies against the B.1.351, B.1.1.7 + E484K, P.1, and B.1.427/B.1.429 variants. The decrease in neutralizing titers was notably lower in the B.1.351 variant [409].

**ChAdOx1 nCoV-19 vaccine:** A two-course [410] unit of the ChAdOx1 nCoV-19 vaccine did not give protection against the B.1.351 variant by low to moderate COVID-19 SARS-CoV-2 vaccines, according to the obtained data from a double-blind, multicenter, randomized control trial with a total of 33725432 participants. Data from another randomized control trial in regard to the ChAdOx1 nCoV-19 vaccine demonstrated that in vitro neutralization activity against the B.1.1.7 variant was decreased in comparison to a non-B.1.1.7 variant, and the clinical effectiveness of the vaccine was 70.4% and 81.5% for B.1.1.7 and non-B.1.1.7 variants, respectively [86,171].

**Figure 15:**
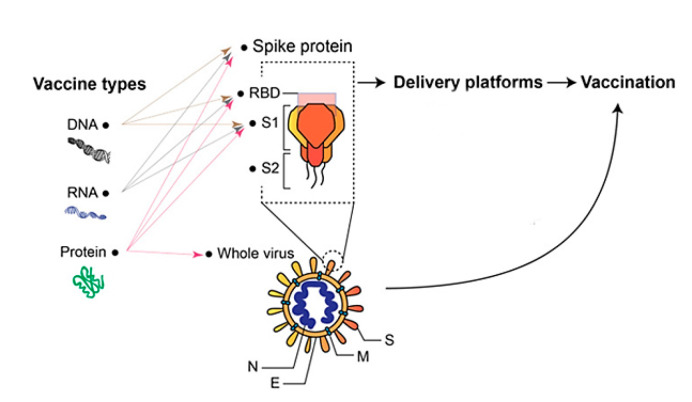
Different targets for vaccine development (picture taken and modified from [114].

The spike (S) protein of SARS-CoV-2 plays a vital role in the receptor recognition and cell membrane fusion process [336]. The fundamental role of the S protein in viral infection indicates that it is a potential target for vaccine development, antibody-blocking therapy, and small-molecule inhibitors (see Figure 15). Vaccines targeting various SARS-CoV-2 proteins are under development [198].

The mutation of the aspartate (D) at position 614 to glycine (G614) results in an intensified infective strain of SARS-CoV-2 [3,411,412], which makes it more difficult to develop antibodies or vaccines that target non-conservative regions.

To effectively prevent disease, combinations of different mAbs that identify different epitopes on the SARS-CoV-2 S surface can be assessed to neutralize a wide range of isolates, including escape mutants [413].

To avoid severe adverse events in safe and effective vaccine development, preclinical trials play a crucial role, which need to be carried out with caution. Furthermore, international organizations i.e., the WHO, CEPI, GAVI, and the Bill and Melinda Gates Foundation, need to be more cooperative with each other to ensure generous funding for vaccine development [140].

### 6.4. Affordability

Self-procuring middle-income countries are buying EUA (Emergency Use Authorization) vaccines with the median value of USD 5.30 (IQR 0.79–18.30), and self-procuring high-income countries are buying EUA vaccines with the median value of USD 16.3 (IQR 6.5–22.0) [153].

Countries covered by Gavi (Global Alliance for Vaccines and Immunization), which is a major buyer of vaccines for low-income countries, have paid the lowest prices per dose median value of USD 0·57 (IQR 0.16–1.90), followed by countries covered by UNICEF with the median value of USD 0.80 (IQR 0.16–2.80) and the Pan American Health Organization (PAHO) with the median value of USD 3.50 (IQR 0.87–13.0). The premarket purchasing costs of EUA vaccines (see Table 12) currently account for pandemic pricing, which might be changing [153].

## 7. Discussion

Preclinical studies of the vaccine MAP have employed animal models ranging from rodents to primates. The MAP approach has been used to test a variety of vaccines, including novel outbreak pandemic vaccines, and has been demonstrated to elicit equal or superior immune responses when compared to IM and other immunization techniques. Clinical tests have been conducted to determine the vaccine MAP’s stability, safety, and immunological efficacy, with good and comparable results when compared to IM administration. However, difficulties such as mass manufacturing, pricing, an aseptic process, and reproducible quality remain. Because vaccine microneedles are an immunization product, the same standards that apply to vaccine manufacturing processes also apply to vaccine microneedle production. As a result, developing the final clinical outcome of vaccine microneedles will take a while. A suitable immunization MAP injector, as well as extra vaccine MAP packaging, are additional cost considerations. Several microneedle manufacturers with large-scale capabilities, on the other hand, have already partnered with vaccine makers to generate vaccine MAPs, with improved immunological results reported. If the restrictions mentioned above are solved, several vaccines will be combined into a microneedle system and given using MAPs. MAPs, as well as syringes and needles, will be used. In the near future, they will be used as a vaccine delivery method.

The benefits of DMN vaccination include dose reduction, painless immunization, and the avoidance of needle injuries. Furthermore, by increasing vaccine stability, lowering vaccine waste, and minimizing the burden on trained personnel, it has the potential to enhance vaccination coverage in disadvantaged countries. Therefore, many more advances in different areas of DMN development are needed before regulatory approval and commercial scale-up can be accomplished.

Fabrication methods must be enhanced even further to ensure minimal antigen loss, which is often promised but seldom verified in the literature and has yet to be shown on a standard laboratory level of output. Analytical challenges include potency testing and stability testing during production and storage, as well as the quantification and repeatability of antigen/adjuvant dose administered in the skin. Furthermore, the relevance of the applicator device should not be disregarded, as it has the potential to standardize DMN administration and vaccine dispersion into the skin, whereas manual application is preferred in logistical situations. DMN immunization exhibited comparable or higher antibody and cellular responses in preclinical studies than conventional vaccinations. Furthermore, cellular immune responses were evoked more strongly by sustained antigen release from nanoparticles or cross-linked structures in DMNs than by fast release from DMNs or liquid solution. While prolonged DMN delivery did not enhance the immune responses any more than rapid DMN release, further study is needed to back up this conclusion. To enhance DMN vaccination, further systematic study, such as the discovery of optimal adjuvants and the evaluation of the impact of DMN geometry, may be necessary in the future. Although the ideal DMN patch has yet to be found, substantial progress has been made. To transform DMNs into products that are safe, effective, inexpensive, and widely used, more testing is needed. To get microneedles on the market, the proper type of microneedle must be selected for (trans)dermal drug delivery (e.g., hollow, solid, geometry, material, density, and length). Microneedle application devices should also be used to provide adequate and reproducible punctures, as well as the ability to self-administer. Consequently, for each kind of vaccine formulation, new microneedle-based solutions for (trans)dermal drug administration should comprise the medicine, a stable vaccine formulation, appropriate microneedles, and a microneedle application device all in one package. Microneedle-based skin vaccination has been proven to be more dose-effective than traditional intramuscular and subcutaneous immunization in both human and animal studies. Various microneedle-based drug-delivery techniques have been used to deliver therapeutic proteins; however, protein structure was only partially explored in the articles listed above. Nothing is known about the immunological side effects of microneedle-based protein delivery. Protein structure preservation is significantly more important for therapeutic proteins than it is for vaccines during manufacture, storage, and use. Any reshaping of novel therapeutics, for example, might diminish drug efficacy while simultaneously compromising safety. However, undesirable immunogenicity may result in the full loss of a protein’s therapeutic effect owing to neutralizing antibodies, the depletion of endogenous proteins, or the collapse of the immune regulation to self-antigens. Because the skin is such a powerful immunologic organ, the latter is perhaps the most severe safety issue. This emphasizes the need to thoroughly analyze protein aggregates and subvisible particles that may be present in and released from transdermal microneedle-based formulations, since they are known to be major risk factors for undesired protein immunogenicity. However, no reports regarding the undesired immunogenicity of therapeutic protein delivery by microneedles have been published to our knowledge, and research in this area is critically needed to advance this field. This necessitates a thorough examination of protein structure in order to ensure its integrity throughout manufacturing and release, as well as immunogenicity tests in appropriate animal models. The microneedle array patch vaccines are still undergoing stability testing, although there is evidence in the literature that vaccine components, including proteins, are stable. Integration into the matrix of the polymers used in the microneedle array patch is usually used to stabilize the product, and as shown by maintenance, maintain their conformational configurations binding role of antibodies. At 25 °C, recombinant adenovirus vaccinations maintained their immunogenicity for at least a month. As a result, the vaccines delivered via the dissolvable microneedles array patch could minimize the expenses associated with the distribution and delivery of vaccines that require extreme low temperatures to maintain their viability e.g., the mRNA-based formulation in the coronavirus vaccines. Overall, microneedle-based (trans)dermal drug delivery may have a significant influence on future medicine, both for COVID-19 vaccination and for therapeutic drug delivery.

## 8. Conclusions

The use of dissolving microneedles for transdermal drug delivery has gained a lot of attention. This is because these devices can include medicines in polymeric matrices that break down in the skin when applied, releasing chemicals that are then delivered into the bloodstream. The requirement to impart mechanical strength while enabling rapid start of action, depending on the individual chemical being delivered, drives the diversity of designs. Micro-molding is the most common manufacturing technology, while drawing lithography and droplet-born air blowing (DAB) are becoming more popular. In this review, the progress achieved in several laboratories on the use of dissolving microneedles for the transdermal administration of a wide range of vaccinations for various illnesses was presented, including the possibility for mass immunization for COVID-19. The greatest advancements are always created at the most critical periods of adversity. Hence, to defeat this pandemic and to keep it under our control, the entire idea of mass vaccination might change due to the implementation of dissolvable microneedles for COVID-19 vaccination. Ideally, this novel vaccine delivery system will have a global outreach, and billions of people will be vaccinated to finally attain herd immunity.

## Figures and Tables

**Table 1:** Comparison of syringe and MN vaccination.

Syringes and Needles (Limitations)	Microneedles in Vaccine Delivery		References
	Strengths	Weaknesses	
Needle use is risky	Painless	Cost of manufacturing is uncertain	[45,64]
Lower thermal stability	Higher vaccine coverage	Production in bulk	[45,65]
Storage and delivery require cold chain	Higher thermal stability during storge	The administration capacity during production	[66,67]
Administration requires expert personnel	Higher shelf life at room temperature	There is still a long way to go before FDA approval is granted and it is commercially available	[51,68,69]
	Allows self-administration		

**Table 2:** SARS-CoV-2-induced immunopathology in humans: symptoms and clinical consequences.

SL No.	Organ System	Clinical Outcomes	Clinical Manifestations	References
1.	Vascular system	Cytokine storm Lymphocyte count Thrombocytopenia-associated mortality ARDS Vasculitis and vascular dysfunction	Elevated IL-6, TNFα, and IL-1β Elevated T helper 17 cells (TH17), plasma cells, CD8+ T cell activity, and decreased regulatory T cells Reduced platelet to lymphocyte ratio Elevated levels of ferritin Elevated VEGF, IL-10, and IL-8	[72,73]
2.	Lungs	Pneumonia with ARDS and dyspnea	Elevated IL-6, TNFα, IL-1β, IL-10, and IL-8	[69,74]
3.	Kidneys	Proteinuria and hematuria	Elevated urea and creatinine levels	[75,76]
4.	Liver	Steatosis and abnormal liver function	Elevated AST, ALT, CRP, and albumin levels	[77,78]
5.	Heart	Acute myocardial injury and chronic CVS damage	Elevated CK and LDH levels	[73,79]
6.	Intestine	Microbial infection, diarrhea, and severe acute ulcerative colitis	Reduced T cell and NK cell count (Lymphopenia)	[75,80]
7.	Brain	Encephalopathy, headache, and ischemic stroke	Elevated CRP, D-dimer, and ferritin levels	[81,82]

Note: NK—natural killer, LDH—lactate dehydrogenase, CRP—C-reactive protein, AST—aspartate transaminase, TNF—tumor necrosis factor, IL—interleukin, ALT—alanine transaminase.

**Table 3:** Vaccine platforms against SARS-CoV-2.

SL No.	Vaccine Technology	Principle	Advantage	Disadvantage	References
1.	mRNA-based	Delivery of modified mRNA	Scalable production Cytoplasmic No vector or foreign DNA Two or more antigens Self-amplifying mRNAs provide sustained expression Cellular and humoral responses	Stringent preparation/storage Less stable Low efficiency of delivery Transient expression (except SAM) Fortuitous immune response Cost	[27,77,156]
2.	DNA-based	Vector-based delivery of a viral gene	Easy to generate Stable Storage at room temperature Scalable Cellular and humoral responses Two or more antigens No adjuvant Ease of delivery Low cost	Issues associated with vector DNA, such as immunogenicity and genomic integration and pre-existing immunity Purity Pathogenicity due to recombination with wild-type virus	[114,157,158]
3.	Peptide-based	A fragment of whole-length viral peptide	Non-infectious Robust immune response Safe Ease of delivery	Challenging manufacturing Stability Need for adjuvant	[159,160]
4.	Live attenuated virus	De-optimization of the genome (to reduce pathogenicity)	Multiple viral antigens Strong immune response	Safety concerns Labor intensive	[157,161]
5.	Inactivated virus	Chemically or UV-inactivated virus	Relatively simple Strong immune response	Risk of partial inactivation Risk of becoming pathogenic	[92,162,163]

**Table 4:** Current mRNA COVID-19 vaccine Stability profile, dose, and dosing schedule.

Stability Profile	Manufacturer		References
	Moderna	Pfizer-BioNTech	
Frozen State	−20 °C up to 6 months	−80 °C to −60 °C up to 6 months	[78,89,109,166,167,168]
2–8 °C	30 days	Up to 5 days	
Room Temperature	Up to 12 h.	Up to 2 h. (up to 6 h. after dilution)	
Dose	100 µg (0.5 mL)	30 µg (0.3 mL)	[109,159,169]
Dosing Schedule	Day 1, Day 29	Day 1, Day 21	

**Table 5:** Extent of utility of DMN in vaccination.

SL No.	Criteria	DMN Array Patch (Score)
1.	Manufacturing cost	**
2.	Mass production	***
3.	Self-administration	*****
4.	Wear time	***
5.	Material biocompatibility	***
6.	Accurate dosage delivery	***
7.	Aseptic process	***
8.	Stability against humidity	**
9.	Waste generation	*****

Note: *****—highest, ***—moderate, **—lowest.

**Table 6:** Model animals and vaccines for dissolving microneedle array patches (DMAP).

SL No.	Types of Vaccine	Animal Model	References
1.	Influenza (inactivated)	Mouse	[245,246]
2.	Hepatitis B (recombinant subunit)	Mouse	[78,159]
3.	HIV (recombinant vector)	Mouse	[247,248]
4.	Dengue virus (live attenuated)	Mouse	[63]
5.	Ebola (DNA)	Mouse	[249,250]
6.	Enterovirus (VLPs)	Mouse	[63]
7.	Rotavirus (inactivated)	Mouse	[67]
8.	Polio virus (inactivated)	Mouse	[128,251]
9.	Streptococcus (inactivated)	Mouse	[63]
10.	Staphylococcus (recombinant subunit)	Mouse	[63,166]
11.	Shigella (BLP)	Mouse	[46,63]
12.	Clostridium (toxoid)	Mouse	[46,63]
13.	BCG (live attenuated)	Mouse	[107,252]
14.	Neisseria gonorrhea (inactivated)	Mouse	[63,229,253,254]
15.	Pseudomonas aeruginosa (inactivated)	Mouse	[64,253,254]
16.	Orientia tsutsugamushi (recombinant subunit)	Mouse	[64,253,254]
17.	Malaria (recombinant subunit)	Mouse	[63,253,255]
18.	Influenza, DT, tetanus toxoid (inactivated)	Rat	[58,256]
19.	BCG (live attenuated)	Mouse	[257]
20.	Influenza (inactivated)	Guinea pig	[40,245]
21.	Hepatitis B (recombinant subunit)	Pig	[258,259]
22.	Hepatitis C (VLPs)	Mouse	[196,260]
23.	Rabies (DNA)	Dog	[63,156]
24.	IPV (inactivated)	Monkey	[261,262]
25.	Measles (live attenuated)	Mouse	[63,128]
26.	Hepatitis B (recombinant subunit)	Mouse	[258,259]
27.	Tetanus toxoid (inactivated)	Pregnant mouse	[256,263]
28.	Measles, rubella (live attenuated)	Infant monkey	[264,265]

Note: IPV stands for inactivated poliovirus vaccine; VLPs stands for virus-like particles; BCG stands for Bacille Calmette–Guerin; HIV stands for human immunodeficiency virus; DT stands for diphtheria and tetanus vaccine; BLP stands for bacterium like particles.

**Table 7:** Research works carried out to establish MN-mediated vaccine delivery.

Targeted Molecule	Disease Causing Microorganisms							
Nucleic Acid	Rabies [330]	BCG [331]	Ebola [250]	Hepatitis B virus [258]		Porcine circovirus type 2 [332]		
Viral Vector	SARS-CoV-2 [142]		Zika [333]	HIV [247,334,335]				
Protein-Based or VLP	SARS-CoV-2 [336]	HIV [337]	Influenza [33,233,245,338,339,340,341,342,343,344,345,346,347,348]	Hepatitis B [259,260,349]	Diphtheria [256,305,350]	HPV [65]	Tetanus [263,306,350]	Malaria [256]
Inactivated or Live Attenuated	Influenza [66,246,351,352,353,354]	Polio virus [261,355]	Rubella [325,356]	Adenovirus [33,357]	Streptococcus [358]	BCG [257]	Measles [325,356,359]	Rotavirus [67]

**Table 8:** Comparison between DMNs and conventional syringes regarding cost reduction.

SL No.	Sections Where Huge Amounts of Costs Can Be Reduced	Dissolving MNs Patch	Conventional Syringes
1.	Maintenance cost of cold chain system for storage after manufacturing vaccine	×	√
2.	Cold chain system cost during distribution of vaccine	×	√
3.	Syringes and vials’ manufacturing cost	×	√
4.	Sharp waste disposal procedure cost	×	√
5.	Plastic and glass waste management cost	×	√
6.	Training cost (training up the caregivers for syringe and vial handling, disposal, etc.)	×	√

Note: √ = yes, and × = no.

**Table 9:** Stability studies of vaccine DMAP.

SL No.	Vaccine	Stabilizer	Temperature	Period	References
1.	Influenza (inactivated)	Trehalose	4 °C, 25 °C, 37 °C	3 months	[40,69]
2.	Influenza (inactivated)	Trehalose	40 °C	6 months	[40,69]
3.	Influenza (inactivated)	Trehalose	35 °C	12 months	[40,69]
4.	Rabies (DNA)	Sucrose	4 °C	3 weeks	[61,67,372]
5.	Hepatitis B (recombinant subunit)	-	4 °C	3 months	[258,260]
6.	Hepatitis B (recombinant subunit)	Sucrose	45 °C	6 months	[258,260]
7.	Influenza (subunit)	Arginine + heptagluconate	25 °C Freeze–thawing	24 months5 cycles	[32,326]
8.	BCG (live attenuated)	-	25 °C	2 months	[252]
9.	Tetanus toxoid/Diphtheria toxoid (divalent subunit)	-	4 °C	24 weeks	[256,263]
10.	Scrub typhus (recombinant subunit)	-	25 °C	4 weeks	[63]

**Table 10:** Analytical methods to determine and monitor quality attributes and stability of mRNA vaccine bulk drug substance and final drug product.

SL No.	Assay	Purpose	References
**1.**	**Characterizing DNA templates and RNA transcripts**		
DNA template sequencing/mRNA sequencing identification of mRNA		Identification of mRNA	[74,159]
UV spectroscopy (A260 nm, A260/A280, A260/A230) Quantification—purity dependent		Quantification—purity dependent	[203]
Fluorescence-based assays (e.g., residual DNA)		Quantification—purity assessment	[13]
Agarose/acrylamide electrophoresis		Molecular mass, RNA integrity, and quantification	[77]
Reverse transcriptase qPCR		Identification and quantification of mRNA	[375]
Blot for dsRNA		Quality assessment	[139]
mRNA capping analysis		Quality assessment	[159]
mRNA polyadenylated tail analysis		Quality assessment	[13]
Chromatographic assays		Quantity and quality assessment	[77]
**2.**	**Characterizing mRNA-encoded translation products**		
In vitro translation—cell-free medium		Translation into target protein	[156]
mRNA evaluation using various cell-based systems		Translation product analysis and potential toxicity assay	[35,91]
**3.**	**Characterizing mRNA-lipid/protein complexes**		
Light scattering		Particle size (distribution)	[72]
(Gel) electrophoresis		Assessing bound/unbound mRNA and surface charge	[107]
Laser Doppler electrophoresis		Zeta potential	[77]
Chromatographic assays/mass spectrometry		Quantification and integrity of carrier lipids/protein	[72]
Fluorescent dyes		Encapsulation efficiency	[372]
**4.**	**General pharmaceutical tests**	Appearance, pH, osmolality, endotoxin concentration, and sterility	[107]

**Table 11:** Companies developing microneedles for vaccine delivery.

SL No.	Company	Type of Microneedle	Disease
1.	Micron Biomedical	Dissolving microneedle	Inactivated rotavirus
2.	3M (Kindeva)	Hollow microneedle	Cancer vaccines
3.	BD Technologies (BS Soluvia)	Stainless steel microneedle	Influenza
4.	Flugen	Metal microneedles	Influenza
5.	Debiotech	Hollow microneedles	COVID-19
6.	Vendari (Vaxipatch)	Stainless steel microneedle	Influenza and COVID-19
7.	Nanopass (Microjet^TM^)	Silicon microneedles	Influenza, Polio, Varicella-Zoster, cancers, Hepatitis B, and COVID-19
8.	BioSeren Tach Inc.	Dissolving microneedles	Vaccine
9.	Sorrento Therapeutics (Solusa)	Nanotopographical imprinted microneedles (coated)	Immuno-oncology
10.	Vaxxas (Nanopatch^TM^)	Coated microneedles array patch	Influenza, COVID-19
11.	Quadmedicine	Dissolving microneedles	Influenza, Canine Influenza
12.	Vaxess	Dissolving microneedles	Influenza, COVID-19, and skin cancer
13.	Raphas	Dissolving microneedles	HPV, Polio, T dap, HBV, IPV, and Hepatitis B

**Table 12:** Overview of leading companies with premarket price range [399].

SL No.	Company	Current R&D Stage	Price Range per Course	Platform Technology
1.	AstraZeneca/Oxford University	Phase II/III	USD 6-USD 8	Non-replicating viral vector
2.	Pfizer/BioNTech		USD 37-USD 39	mRNA
3.	Johnson & Johnson/Janssen	Phase III	USD 20	Non-replicating viral vector
4.	Moderna		USD 30-USD 74	mRNA
5.	Novavax		USD 6-USD 32	Protein subunit
6.	SinoVac		Undisclosed	Inactivated

## Data Availability

There are no additional raw data for this paper. The paper only uses secondary data from published papers, and all credits to these data have been made via citations and copyright permissions.
